# The interaction between serum uric acid and triglycerides level on blood pressure in middle-aged and elderly individuals in China: result from a large national cohort study

**DOI:** 10.1186/s12872-020-01468-3

**Published:** 2020-04-15

**Authors:** Lin Zhang, Jin-long Li, Lei-lei Guo, Hong Li, Dan Li, Guang Xu

**Affiliations:** 1 Department of Surgical Nursing, School of Nursing, Jinzhou Medical University, No.40, Section 3, Songpo Road, Linghe District, Jinzhou City, Liaoning Province People’s Republic of China; 2 Department of Occupational and Environmental Health, Key Laboratory of Occupational Health and Safety for Coal Industry in Hebei Province, School of Public Health, North China University of Science and Technology, Tangshan, Hebei Province People’s Republic of China; 3 Experimental Center for Nursing, School of Nursing, Jinzhou Medical University, No.40, Section 3, Songpo Road, Linghe District, Jinzhou City, Liaoning Province People’s Republic of China; 4 Department of Radiotherapy, Third Affiliated Hospital of Jinzhou Medical University, No.2, Section 5, Heping Road, Linghe District, Jinzhou City, Liaoning Province People’s Republic of China

**Keywords:** Blood pressure, Middle-aged and elderly individuals, Serum uric acid, Triglycerides

## Abstract

**Background:**

The purpose of the research was to explore the extent of interaction between triglycerides (TG) and serum uric acid (SUA) level with blood pressure (BP) in middle-aged and elderly individuals in China.

**Methods:**

Data were selected from the China Health and Retirement Longitudinal Study (CHARLS), a cross-sectional study. 3345(46.99%) men with average ages of 60.24 ± 9.24 years and 3774 (53.01%) women with average ages of 59.91 ± 9.95 years were included in the study. Differences between gender, or between categories of blood pressure levels were evaluated by t-test or chi-square test. The adjusted associations between various characteristics and BP status were first compared using linear regression models, as appropriate. Then, A general linear model adjusted for confounding factors (socio-demographic characteristics [age, educational levels, marital status, place of residence], health behaviors [cigarette smoking, alcohol drinking, eating habits, social and leisure activities, accidental injury, physical activities], medical history [history of cardiovascular diseases, hepatitis history, antidiabetic drugs, history of antilipidemic medication, anti-hypertensive therapy], metabolic measures [C-reactive protein (CRP), hemoglobin A1c (HbA1c), fasting plasma glucose (FPG), low-density lipoprotein cholesterol (LDL-C), high-density lipoprotein cholesterol (HDL-C), estimated glomerular filtration rate (eGFR), body mass index (BMI)]) was used to examine the synergistic effect of SUA and TG level on BP in middle-aged and elderly individuals in China.

**Results:**

Age-adjusted partial Pearson’s correlation coefficient showed that SUA and TG level positively correlated with both systolic blood pressure (SBP) and diastolic blood pressure (DBP) in both men and women. Multiple linear regression analysis showed the TG level was significantly and positively associated with SBP and DBP in both men (SBP: β =0.068, *P* = 0.001; DBP: β =0.064, *P* = 0.002) and women (SBP: β =0.061, *P* = 0.002; DBP: β =0.084, *P* = 0.000), but SUA were significantly and positively associated with SBP in both men (SBP: β =0.047, *P* = 0.013) and women (SBP: β =0.040, *P* = 0.028), regardless of other confounding factors. After adjusting for related potential confounders, evidence of interaction between SUA and TG level on SBP (men: β = − 1.090, *P* = 0.726; women: β = − 0.692, *P* = 0.861) and DBP (men: β = − 1.026, *P* = 0.572; women: β = − 0.794, *P* = 0.842) was not observed.

**Conclusion:**

The interaction effect of SUA and TG level on BP was not observed in our study. Moreover, high SUA level was significantly associated with SBP, while high TG level was strongly related to both DBP and SBP.

## Background

As well known, according to the relationship between prehypertension and cardiovascular diseases [1–3] and the etiologies of cardiovascular diseases (CVDs) [4–6], the hypertension is defined with the standards as follows: ①diastolic blood pressure (DBP) of > 90 mmHg; and/or ②systolic blood pressure (SBP) of > 140 mmHg. Hypertension is a cluster of risk factors [7–14] associated with ageing, central obesity, overweight, the household heredity factors, unhealthy behavior and lifestyles (cigarette smoking, alcohol consumption, and lack of physical activities), diabetes, dyslipidemia, low levels of high-density lipoprotein cholesterol (HDL-C), high levels of low-density lipoprotein cholesterol (LDL-C), elevated fasting glucose levels, and elevated triglycerides (TG). Hypertension is serious complex, and patients suffer from the physical, psychosocial, and economic burden, it has become serious public health worldwide [15]. Recently, the prevalence and incidence of hypertension remained higher up in China [16, 17]. For the ageing population increases, it is disproportionately high among middle-aged and elderly individuals in China [18–20]. Hypertension is a multi-factor caused disease. Recently, hypertension has become crucial for public health worldwide. In sum, hypertension prevention and treatment strategies, and its risks should be carefully studied. Exploring its timely associated risks and their interaction of hypertension may provide insight into public health implications for the prevention and management of hypertension in future.

Serum uric acid (SUA) is an endogenous end product and is involved in the production of reactive oxygen species. It is crucial to evaluate its status in advance of chronic disease development [21]. In recent years, as a critical mark, systemic measured by SUA has become an essential marker for chronic disease development. Studies have conducted that SUA is associated with various diseases, such as CVDs [22–25], prehypertension [26–29], metabolic syndrome [30–32], and hypertension [33–35]. However, despite the association between SUA level and these risk conditions, SUA level may not be regarded as an independent risk factor. Since SUA level is highly associated with overweight, obesity and other risk factors [36–38], which is in turn associated with risk of hypertension, a causal condition may exist between TG and risk of hypertension. Therefore, the association between SUA level and risk of hypertension and the effects of TG on this association are of considerable interest, and a modulating effect between TG and SUA level on blood pressure (BP) may also be fully considered.

To date, few studies on the association and interaction analysis between SUA and TG level and BP were conducted in individuals aged ≥45 years. Thus, this study aimed to determine the prevalence of normotension and hypertension and their association with SUA, TG level, and other confounding factors based on gender using the individuals aged ≥45 years from cross-sectional study data (CHARLS) in China.

## Methods

### Study design and setting

**Fig. 1:**
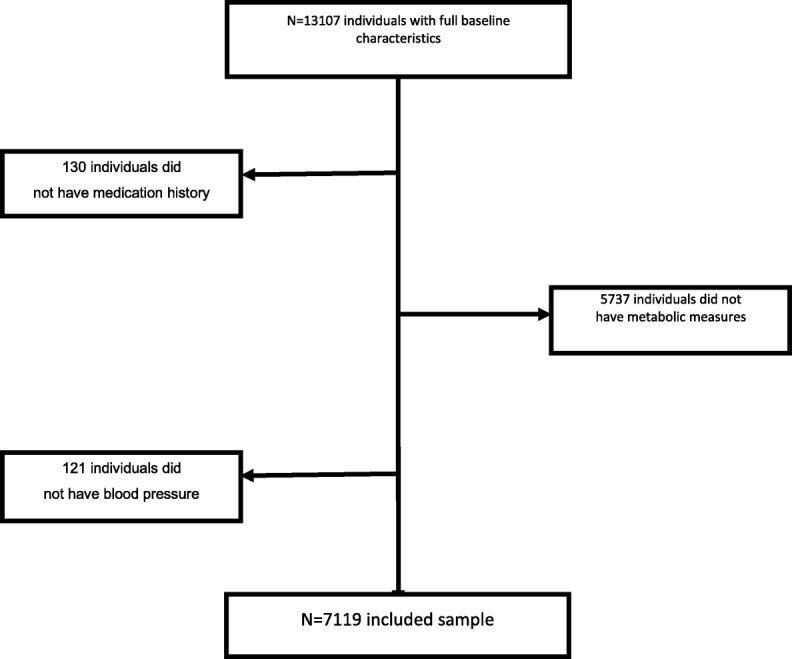
Selection of participants

Data from the CHARLS were used in our study. The CHARLS was a nationally representative longitudinal study conducted by the China Centre for Economic Research at Peking University [39]. In the 2011 CHARLS Wave1, at baseline, 13107 individuals were recruited for a longitudinal study, 130 individuals were excluded because the absence of medication history, a group of 5737 participants did not have their metabolic measures, and 121 individuals did not have their blood pressure. Finally, 7119 individuals were included in the analyses. Figure 1 summarized the selection of participants.

### Participants

The participants of the study were from the CHARLS, Wave 1 (2011) [39]. The CHARLS involved 7119 individuals ≥45 years old, out of whom 46.99% were 60.24 ± 9.24 years and 53.01% women were 59.91 ± 9.95 years.

### Self-reported factors

Variables like age, educational levels (four categories, illiterate, less than elementary school, high school, and above vocational school), marital status (dichotomous variables, the single and married), place of residence (dichotomous variables, rural and urban), cigarette smoking (three categories, no, former smoke, and current smoke), alcohol consumption (three categories, no, less than once a month, and more than once a month), eating habit (three categories, ≤two meals per day, three meals per day, and ≥ four meals per day), social and leisure activities (dichotomous variables, no and yes), accidental injury (dichotomous variables, no and yes), physical exercise (three categories, no physical exercise, less than physical exercises, and regular physical exercises), history of liver disease (dichotomous variables, no and yes), history of cardiovascular diseases (dichotomous variables, no and yes), antidiabetic medication (dichotomous variables, no and yes), antilipidemic medication (dichotomous variables, no and yes), and antihypertensive medication (dichotomous variables, no and yes) were obtained using a self-reported questionnaire, and most variables based on our previous studies [40–44].

### Measurements

BMI was calculated based on the measured weight and height of the participants [45]. CRP was measured using immunoturbidimetric assay. FPG, TG, LDL-C, HDL-C, and HbA1c were analyzed using the enzymatic colorimetric tests, SUA level were analyzed using the urinalysis (UA) plus method. The mean of the three measurements determined the average value of BP. Estimated glomerular filtration rate (eGFR) was measured by the chronic kidney disease epidemiology collaboration (CKD-EPI) creatinine-cystatin equations [46]. TG was divided into two categories: < 150 mg/dL and ≥ 150 mg/dL. Hyperuricemia (HUA) was defined as SUA concentration of > 7 mg/dL in men and > 6 mg/dL in women [47]. Participants were divided into hypertension (defined as SBP of ≥140 mmHg and/or DBP of ≥90 mmHg), and normotension (defined as not being on antihypertensive therapies with an SBP of < 140 mmHg and DBP of < 90 mmHg) groups, the categorization has been widely used in previous studies [41, 44].

### Statistical analysis

Data were analyzed by using SPSS17.0 software for Windows10 (IBM Corp., Armonk, NY, USA) and expressed as the mean SD or frequency, as appropriate. Differences between gender, or between categories of blood pressure levels were evaluated by t-test or chi-square test. The adjusted associations between various characteristics and BP status were first compared using linear regression models, as appropriate. Then, general linear models adjusting for related potential confounders were used to examine the synergistic effect of SUA and TG level on blood pressure in middle-aged and elderly individuals in China. 2-tailed and a value of *P* of 0.05 were considered statistically significant.

## Results

In total, 7119 participants who effectively completed the questionnaires were included in our research. The baseline of demographic variables was shown in Table 1, and most variables based on our previous studies [40–44]. Overall, 3345(46.99%) of the participants were men, and 3774 (53.01%) of the participants were women. The average ages of the men and women were 60.24 ± 9.24 and 59.91 ± 9.95 years, respectively. In the men, the mean and standard deviation of SUA level were 4.87 ± 1.24 mg/dl in the normotensive group, and 5.20 ± 1.33 mg/dl in the hypertensive group, respectively. In the men, the mean and standard deviation of TG level were 120.58 ± 100.65 mg/dl in the normotensive group, and 133.51 ± 111.81 mg/dl in the hypertensive group, respectively. In the women, the mean and standard deviation of SUA level were 3.93 ± 1.04 mg/dl in the normotensive group, and 4.24 ± 1.16 mg/dl in the hypertensive group, respectively. In the women, the mean and standard deviation of TG level were 129.26 ± 82.88 mg/dl in the normotensive group, and 154.49 ± 112.79 mg/dl, respectively. Table 1 showed the relationship between various characteristics and BP levels in the participants. Significant differences in distribution were observed between blood pressure status in the men in all of the variables, except cigarette smoking, alcohol consumption, eating habit, social and leisure activities, physical exercise, hepatitis history, anti-diabetic medication, and HDL-C. Age, CRP, HbA1c, FPG, LDL-C, BMI, TG, SUA, SBP, and DBP were significantly higher in hypertension than those in the normotension, whereas, eGFR level were lower in hypertension than that in the normotension. Significant differences in distribution were observed between blood pressure status in women in all of the variables, except the place of residence, cigarette smoking, eating habit, social and leisure activities, accidental injury, regular physical exercises, and hepatitis history. Age, CRP, HbA1c, FPG, LDL-C, BMI, TG, SBP, SUA, and DBP were significantly higher in hypertension than those in the normotension. However, HDL-C and eGFR level were lower in hypertension than that in the normotension.

**Table 1:** Baseline of demographic variables of participants categorized by gender and blood pressure status in men and women(*N* = 7119)

Variables	Men(***n*** = 3345)		t/χ^2^	***P***	Women (***n*** = 3774)		t/χ^2^	***P***
	Normotension (***n*** = 2267)	Hypertension (***n*** = 1078)			Normotension (***n*** = 2492)	Hypertension (***n*** = 1282)		
Age (years)	60.5 ± 9.55	63.41 ± 9.53	−8.231	0.000	58.07 ± 9.19	63.5 ± 10.39	−16.433	0.000
Educational levels								
Illiterate	311 (65.20)	166 (34.80)	8.758	0.033	1025 (60.51)	669 (39.49)	45.920	0.000
Less than elementary school	1661 (67.91)	785 (32.09)			1267 (69.54)	555 (30.46)		
High school	197 (74.34)	68 (25.66)			142 (79.78)	36 (20.22)		
Above vocational school	98 (62.42)	59 (37.58)			58 (72.50)	22 (27.50)		
Marital status								
Single	194 (56.73)	148 (43.27)	21.288	0.000	345 (53.41)	301 (46.59)	55.387	0.000
Married	2073 (69.03)	930 (30.97)			2147 (68.64)	981 (31.36)		
Place of residence								
Rural	1553 (69.77)	673 (30.23)	12.108	0.001	1591 (66.60)	798 (33.40)	0.930	0.335
Urban	714 (63.81)	405 (36.19)			901 (65.05)	484 (34.95)		
Cigarette smoking								
No	1320 (68.18)	616 (31.82)	0.395	0.821	2292 (66.36)	1162 (33.64)	2.105	0.349
Former smoke	384 (66.90)	190 (33.10)			51 (60.71)	33 (39.29)		
Current smoke	563 (67.43)	272 (32.57)			149 (63.14)	87 (36.86)		
Alcohol consumption								
No	1026 (67.37)	497 (32.63)	0.686	0.710	2172 (65.44)	1147 (34.56)	6.263	0.044
Less than once a month	239 (69.68)	104 (30.32)			135 (74.18)	47 (25.82)		
More than once a month	1002 (67.75)	477 (32.25)			185 (67.77)	88 (32.23)		
Eating habit								
≤ 2 meals per day	32 (66.67)	16 (33.33)	3.447	0.178	35 (67.31)	17 (32.69)	0.676	0.713
3 meals per day	1945 (68.39)	899 (31.61)			2125 (66.26)	1082 (33.74)		
≥ 4 meals per day	290 (64.02)	163 (35.98)			332 (64.47)	183 (35.53)		
Social and leisure activities								
No	1106 (67.27)	538 (32.73)	0.367	0.545	1250 (66.35)	634 (33.65)	0.169	0.681
Yes	1161 (68.25)	540 (31.75)			1242 (65.71)	648 (34.29)		
Accidental injury								
No	314 (73.02)	116 (26.98)	6.228	0.013	181 (70.7)	75 (29.30)	2.673	0.102
Yes	1953 (67.00)	962 (33.00)			2311 (65.69)	1207 (34.31)		
Physical exercises								
No physical exercise	435 (65.81)	226 (34.19)	1.471	0.479	1492 (64.73)	813 (35.27)	4.543	0.103
Less than regular physical exercises	417 (68.47)	192 (31.53)			483 (67.74)	230 (32.26)		
Regular physical exercises	1415 (68.19)	660 (31.81)			517 (68.39)	239 (31.61)		
History of cardiovascular disease								
No	2065 (68.97)	929 (31.03)	14.634	0.000	2187 (67.46)	1055 (32.54)	13.740	0.000
Yes	206 (58.69)	145 (41.31)			314 (59.02)	218 (40.98)		
Hepatitis history								
No	2170 (67.77)	1032 (32.23)	0.914	0.339	2413 (66.18)	1233 (33.82)	2.532	0.112
Yes	102 (71.33)	41 (28.67)			93 (72.66)	35 (27.34)		
Antilipidemic therapy								
No	2174 (68.13)	1017 (31.87)	4.029	0.045	2388 (67.14)	1169 (32.86)	33.645	0.000
Yes	93 (60.39)	61 (39.61)			104 (47.93)	113 (52.07)		
Antidiabetic drugs								
No	2195 (67.96)	1035 (32.04)	1.454	0.228	2395 (66.51)	1206 (33.49)	8.021	0.005
Yes	72 (62.61)	43 (37.39)			97 (56.07)	76 (43.93)		
Anti-hypertensive therapy								
No	2187 (69.49)	960 (30.51)	72.177	0.000	2386 (67.31)	1159 (32.69)	42.364	0.000
Yes	80 (40.40)	118 (59.60)			106 (46.29)	123 (53.71)		
C-reactive protein (mg/l)	5.21 ± 0.68	5.29 ± 0.85	−2.966	0.003	5.27 ± 0.81	5.37 ± 0.95	−3.648	0.000
HbA1c (%)	2.85 ± 7.4	3.46 ± 8.74	−2.110	0.035	2.12 ± 4.98	3.10 ± 7.17	−4.922	0.000
Fasting plasma glucose (mg/dl)	108.35 ± 32.73	113.93 ± 39.51	−4.304	0.000	108.43 ± 34.39	115.26 ± 44.62	−5.209	0.000
Low density lipoprotein (mg/dl)	111.23 ± 33.92	115.58 ± 35.82	−3.408	0.001	119.98 ± 33.79	122.7 ± 38.35	−2.239	0.025
High density lipoprotein (mg/dl)	51.22 ± 16.17	50.19 ± 16.35	1.719	0.086	52.2 ± 14.16	50.15 ± 14.4	4.205	0.000
eGFR (ml/min/1.73m^2^)	84.26 ± 17.04	79.00 ± 18.48	8.122	0.000	87.63 ± 17.01	80.39 ± 18.51	12.016	0.000
Body mass index (kg/m^2^)	22.52 ± 3.64	23.65 ± 3.69	−8.372	0.000	23.59 ± 3.89	24.67 ± 4.5	−7.653	0.000
Systolic blood pressure (mmHg)	119.73 ± 11.55	155.77 ± 22.4	−61.346	0.000	118.93 ± 11.82	159.23 ± 31.47	−56.586	0.000
Diastolic blood pressure (mmHg)	71.23 ± 9.30	88.88 ± 12.55	−45.640	0.000	71.27 ± 9.05	86.79 ± 11.9	−44.687	0.000
Serum uric acid (mg/dl)	4.87 ± 1.24	5.20 ± 1.33	− 7.077	0.000	3.93 ± 1.04	4.24 ± 1.16	−8.407	0.000
Triglycerides (mg/dl)	120.58 ± 100.65	133.51 ± 111.81	−3.349	0.001	129.26 ± 82.88	154.49 ± 112.79	−7.798	0.000

Table 2 showed the age-adjusted relationship between the baseline of demographic variables and BP status of participants categorized by gender. In the men, firstly, age-adjusted partial Pearson’s correlation coefficient showed that marital status, place of residence, accidental injury, history of CVDs, antilipidemic medication, anti-hypertensive medication, HbA1c, FPG, LDL-C, HDL-C, eGFR, BMI, SUA and TG level significantly correlated with both SBP and DBP. Secondly, anti-diabetic medication positively correlated with SBP. Lastly, alcohol consumption and eating habits significantly correlated with DBP. In the women, firstly, age-adjusted partial Pearson’s correlation coefficient showed that alcohol consumption, history of CVDs, antilipidemic medication, anti-hypertensive medication, anti-diabetic medication, HbA1c, FPG, HDL-C, BMI, SUA and TG level significantly correlated with both SBP and DBP. Secondly, marital status positively correlated with SBP. Thirdly, eating habit, hepatitis history, LDL-C, and eGFR significantly correlated with DBP.

**Table 2:** Age-adjusted relationship between baseline of demographic variables and blood pressure status of participants categorized by gender (*N* = 7119)

Variables	Men (***n*** = 3345)		Women (***n*** = 3774)	
	Systolic blood pressure partial r(*P*-value)	Diastolic blood pressure partial r(*P*-value)	Systolic blood pressure partial r(*P*-value)	Diastolic blood pressure partial r(*P*-value)
Educational levels	0.003 (0.873)	0.028 (0.113)	−0.029 (0.077)	− 0.022 (0.180)
Marital status	0.064 (0.000)	0.050 (0.004)	0.045 (0.007)	0.010 (0.529)
Place of residence	0.080 (0.000)	0.083 (0.000)	0.024 (0.138)	0.014 (0.394)
Cigarette smoking	0.009 (0.590)	− 0.028 (0.107)	− 0.007 (0.656)	0.003 (0.858)
Alcohol consumption	0.017 (0.334)	0.044 (0.011)	−0.052 (0.002)	−0.045 (0.007)
Eating habit	−0.028 (0.107)	−0.047 (0.008)	− 0.011 (0.521)	− 0.052 (0.002)
Social and leisure activities	− 0.009 (0.589)	− 0.002 (0.928)	0.014 (0.380)	0.009 (0.585)
Accidental injury	−0.041 (0.019)	−0.043 (0.013)	− 0.026 (0.115)	−0.008 (0.627)
Physical exercises	0.020 (0.245)	0.001 (0.942)	−0.010 (0.546)	0.006 (0.735)
History of cardiovascular disease	0.050 (0.005)	0.064 (0.000)	0.039 (0.018)	0.039 (0.019)
Hepatitis history	−0.014 (0.409)	−0.009 (0.622)	− 0.020 (0.235)	−0.039 (0.019)
Antilipidemic therapy	0.036 (0.039)	0.046 (0.009)	0.063 (0.000)	0.076 (0.000)
Antidiabetic drugs	0.049 (0.005)	0.023 (0.187)	0.073 (0.000)	0.033 (0.047)
Anti-hypertensive therapy	0.152 (0.000)	0.147 (0.000)	0.106 (0.000)	0.084 (0.000)
C-reactive protein (mg/l)	0.024 (0.177)	0.007 (0.704)	0.030 (0.070)	0.022 (0.172)
HbA1c (%)	0.064 (0.000)	0.070 (0.000)	0.048 (0.004)	0.073 (0.000)
Fasting plasma glucose (mg/dl)	0.093 (0.000)	0.078 (0.000)	0.040 (0.015)	0.082 (0.000)
Low density lipoprotein (mg/dl)	0.083 (0.000)	0.065 (0.000)	0.022 (0.182)	0.034 (0.039)
High density lipoprotein (mg/dl)	−0.054 (0.002)	−0.048 (0.006)	−0.085 (0.000)	− 0.111 (0.000)
eGFR (ml/min/1.73m^2^)	−0.090 (0.000)	− 0.045 (0.009)	− 0.023 (0.156)	−0.042 (0.010)
Body mass index (kg/m^2^)	0.218 (0.000)	0.221 (0.000)	0.165 (0.000)	0.221 (0.000)
**Serum uric acid (mg/dl)**	**0.122 (0.000)**	**0.096 (0.000)**	**0.084 (0.000)**	**0.102 (0.000)**
**Triglycerides (mg/dl)**	**0.111 (0.000)**	**0.109 (0.000)**	**0.103 (0.000)**	**0.143 (0.000)**

**Fig. 2:**
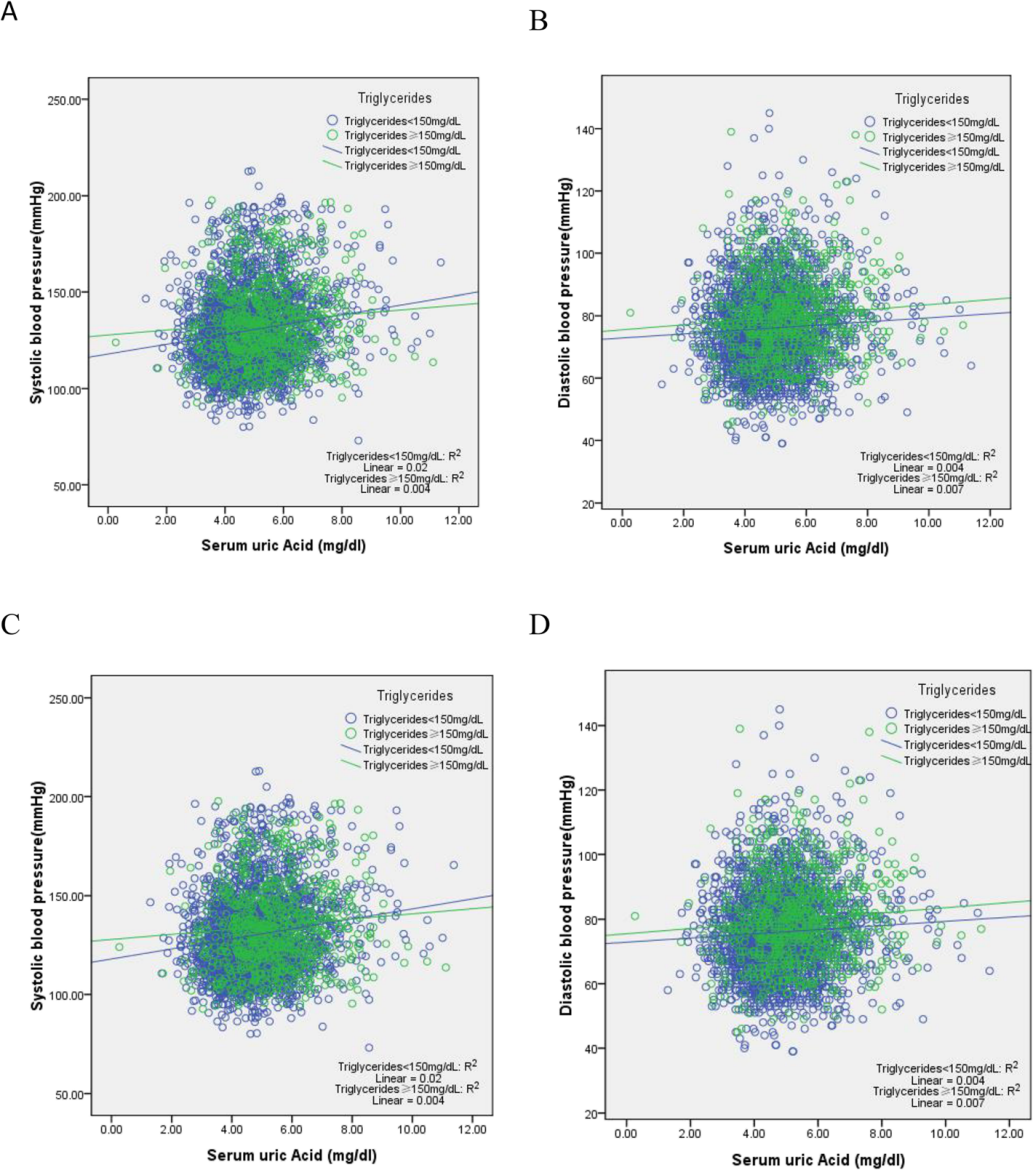
**a** and **b**, correlation between serum uric acid (SUA) and blood pressure (BP) of individuals categorized by triglycerides (TG) in men. **c** and **d**, correlation between serum uric acid (SUA) and blood pressure (BP) of individuals categorized by triglycerides (TG) in women

Table 3 showed a multivariate-adjusted relationship between the baseline of demographic variables and BP in participants categorized by gender. The results showed that the TG level were significantly and positively associated with SBP and DBP in both men (SBP: β =0.068, *P* = 0.001; DBP: β =0.064, *P* = 0.002) and women (SBP: β =0.061, *P* = 0.002; DBP: β =0.084, *P* = 0.000), but SUA were significantly and positively associated with SBP in both men (SBP: β =0.047, *P* = 0.013) and women (SBP: β =0.040, *P* = 0.028), regardless of other confounding factors. We also take care of the direct associations between TG category and SUA levels on BP levels. Figure 2 showed that the lines differenced from others in each figure.

**Table 3:** Multivariate-adjusted relationship between baseline of demographic variables and blood pressure status of participants categorized by gender (*N* = 7119)

Variables	Men (***n*** = 3345)		Women (***n*** = 3774)	
	Systolic blood pressure β(*P*-value)	Diastolic blood pressure β (*P*-value)	Systolic blood pressure β (*P*-value)	Diastolic blood pressure β (*P*-value)
Age (years)	0.144 (0.000)	−0.119 (0.000)	0.224 (0.000)	−0.057 (0.011)
Educational levels	–	–	−0.043 (0.014)	− 0.036 (0.042)
Marital status	0.081 (0.000)	0.070 (0.000)	0.058 (0.001)	–
Place of residence	0.049 (0.004)	0.052 (0.003)	–	–
Cigarette smoking	0.048 (0.005)	–	–	–
Alcohol consumption	–	0.047 (0.008)	−0.036 (0.023)	–
Eating habit	–	−0.055 (0.001)	–	− 0.064 (0.000)
Social and leisure activities	−0.034 (0.041)	–	–	–
Accidental injury	−0.034 (0.036)	− 0.039 (0.019)	–	–
Physical exercises	–	–	–	–
History of CVD	–	0.034 (0.047)	–	–
Hepatitis history	–	–	–	−0.039 (0.015)
Antilipidemic therapy	–	–	–	0.036 (0.034)
Antidiabetic drugs	–	−0.043 (0.021)	0.050 (0.004)	–
Anti-hypertensive therapy	0.126 (0.000)	0.124 (0.000)	0.097 (0.000)	0.075 (0.000)
C-reactive protein (mg/l)	–	–	–	–
HbA1c (%)	–	–	–	–
Fasting plasma glucose (mg/dl)	0.052 (0.017)	–	–	–
Low density lipoprotein (mg/dl)	0.068 (0.000)	0.050 (0.004)	–	–
High density lipoprotein (mg/dl)	–	–	–	–
eGFR (ml/min/1.73m^2^)	−0.091 (0.000)	−0.048 (0.035)	–	−0.045 (0.043)
Body mass index (kg/m^2^)	0.193 (0.000)	0.194 (0.000)	0.129 (0.000)	0.193 (0.000)
Serum uric acid (mg/dl)	**0.047 (0.013)**	–	**0.040 (0.028)**	–
Triglycerides (mg/dl)	**0.068 (0.001)**	**0.064 (0.002)**	**0.061 (0.002)**	**0.084 (0.000)**
R^2^	0.132 (0.000)	0.106 (0.000)	0.120 (0.000)	0.081 (0.000)

A general linear model adjusted for the related confounding factors (socio-demographic characteristics [age, educational levels, marital status, place of residence], health behaviors [smoking habit, alcohol consumption, eating habits, social and leisure activities, accidental injury, physical activities], medical history [history of CVDs, hepatitis history, antidiabetic drugs, anti-hypertensive therapy, history of antilipidemic medication], metabolic measures [CRP, HbA1c, FPG, HDL-C, eGFR, LDL-C, BMI]) was used to explore the combined relationship between SUA and TG level. Evidence of interaction between SUA and TG level on SBP (β = − 1.090, *P* = 0.726 in men; β = − 0.692, *P* = 0.861 in women) and DBP (β = − 1.026, *P* = 0.572 in men; β = − 0.794, *P* = 0.842 in women) was not observed (Table 4).

**Table 4:** Interaction between Triglycerides and uric acid on blood pressure status in Men and Women (*N* = 7119)

Characteristics	Men (***n*** = 3345)		Women (***n*** = 3774)	
	Systolic blood pressure β(*P*-value)	Diastolic blood pressure β(*P*-value)	Systolic blood pressure β(*P*-value)	Diastolic blood pressure β(*P*-value)
Age (years)	0.350 (0.000)	−0.159 (0.000)	0.692 (0.000)	0.711 (0.000)
Educational levels	–	–	−1.694 (0.013)	−1.740 (0.012)
Marital status	6.149 (0.000)	2.975 (0.000)	4.244 (0.001)	–
Place of residence	2.141 (0.009)	1.444 (0.002)	–	–
Cigarette smoking	1.432 (0.002)	–	–	–
Alcohol consumption	–	0.753 (0.001)	−1.911 (0.016)	–
Eating habit	–	−1.917 (0.001)	–	−1.53 (0.196)
Social and leisure activities	−1.681 (0.027)		–	–
Accidental injury	−2.445 (0.030)	−1.737 (0.008)	–	–
Physical exercises	–	–	–	–
History of CVD	–	1.508 (0.039)	–	−3.082 (0.198)
Hepatitis history	–	–	–	4.841 (0.011)
Antilipidemic therapy	–	–	–	
Antidiabetic drugs	–	−1.691 (0.169)	6.722 (0.001)	11.466 (0.000)
Anti-hypertensive therapy	12.729 (0.000)	7.142 (0.000)	11.542 (0.000)	–
C-reactive protein	–	–	–	–
HbA1c (%)	–	–	–	–
Fasting plasma glucose (mg/dl)	0.034 (0.002)	–	–	–
Low density lipoprotein (mg/dl)	0.042 (0.000)	0.019 (0.003)	–	–
High density lipoprotein (mg/dl)	–	–	–	–
eGFR (ml/min/1.73m^2^)	−0.131 (0.000)	−0.032 (0.041)	–	−0.040 (0.200)
Body mass index (kg/m^2^)	1.176 (0.000)	0.686 (0.000)	0.975 (0.000)	0.984 (0.000)
Serum uric acid (0 = ≤7 mg/dL in men and ≤ 6 mg/dL in women, 1= > 7 mg/dL in men and > 6 mg/dL in women)	−2.252 (0.353)	−1.723 (0.223)	−2.424 (0.391)	−1.907 (0.508)
Triglycerides (0 = < 150 mg/dL 1 = ≥150 mg/dL)	− 1.695 (0.571)	−0.560 (0.748)	− 2.387 (0.535)	−2.272 (0.558)
Serum uric acid * Triglycerides	**− 1.090 (0.726)**	**−1.026 (0.572)**	**− 0.692 (0.861)**	**−0.794 (0.842)**

## Discussion

At present, the association of SUA and TG level and the level with BP varied in middle-aged and elderly individuals. In the research, we attempted to determine the hypertension prevalence and its association with TG and SUA level. The results showed that prevalence of hypertension was 32.23% (1078/3345) in men and 33.97% (1282/3774) in women, which is similar to those of the English individuals (men, 36.8%; women, 38.6%) [48]. Moreover, SUA and TG level were significantly (positively) associated with DBP and SBP in both men and women. In general, this finding is consistent with most previous studies [41, 49] that suggested the high SUA and TG level were risk factors for hypertension.

Although previous studies [45, 50, 51] have explored the association and/or interaction analysis between BMI and SUA levels and BP, no consistent results are pointing to such associations. Lee et al. [45], using data from 45,098 Koreans who underwent health examinations at Korea Association of Health Promotion with no history of taking drugs related with UA and/or BP, found that SUA levels were positively associated with SBP and DBP in men aged < 40 years after adjustment for age, diabetes, dyslipidemia, BMI, and eGFR; However, no significant associations were found in men aged 60 years or older. Lyngdoh et al. [50], assessing 549 individuals aged 19–20 years, found that SUA levels tended to be positively associated with DBP and SBP in men. Moreover, the strength of the SUA-BP association was similar in women. Kawamoto et al. [51] found that increased SUA levels were positively associated with BP in participants with BMI < 21.0 kg/m^2^, while there was a negative association between SUA levels, BP in those with BMI ≥ 21.0 kg/m^2^, in whom the interaction between BMI and SUA levels was a significant and independent determinant of both SBP and DBP. The difference between those studies may due to the different sampling methods, the different population, and different confounding variables by controlling. It is interesting to note that only one study [47] reported the interaction between SUA and TG on blood pressure. Furthermore, it was found that there was a significant joint effect of TG and SUA level on DBP. However, in our study, we found no significant interaction between SUA level and TG in relation to blood pressure. A community-based study of a consecutive sample of 3065 individuals suggested that the SUA level was highest in people with abnormal levels of TG [52]. In another study, the association of TG and SUA had been persistent after full adjustment, suggesting that TG correlated independently with SUA levels [53]. The mechanisms that lead to hypertension in participants with high TG or SUA level have not been elucidated. Several studies [54–56] reported that high SUA levels induced endothelial dysfunction through vascular resistance in insulin-induced NO production, potentially leading to hypertension. Additionally, high TG levels are also strongly correlated with insulin resistance, and insulin resistance promotes the development of hypertension by augmenting sympathetic nervous system reactivity, activating the renin-angiotensin system, and stimulating renal tubular sodium reabsorption. Renin-angiotensin system activation is induced not only by high SUA levels but also by high TG levels, and the two factors may have interactive effects on blood pressure. Moreover, SUA is strongly associated with inflammation [57–59], oxidative stress [60–62] and other risk factors for CVDs, such as BMI, TG, and FPG [51]. Risk factors associated with hypertension may lead to decreased vasomotor reactivity, endothelial dysfunction, and arterial stiffness [51], ultimately causing hypertension. Those studies may provide insights into the pathogenic mechanism by which SUA or TG induces hypertension. Though the joint effects were not examined, we found that SUA level was independently associated with SBP in both men and women, and TG independently associated with BP in both men and women. Our study suggested that SUA may play an essential role in SBP, and gender-specific factors may also be crucial. The SUA level was higher in men than in women, which can partially explain the underlying mechanisms that possibly account for gender differences, such as alcohol consumption, whose prevalence is usually higher in men. Additionally, body fat and steroid hormones, and their interaction in middle-aged and older adults may also be associated with hypertension.

This study has three limitations. First, the association and interaction between SUA and TG level and BP become seriously more complex. We only considered the identified confounders. However, some unknown factors still existed. Second, the relationship should be studied prospectively. However, our study investigated the interaction between SUA and TG and BP level in participants aged ≥45 years in a cross-sectional study. More follow-up cohort studies will be needed to determine the associations in the next phase. Thirdly, an investigator-based interview was compared with a self-report questionnaire-based directly on that interview. Finally, more research is needed to confirm the results. However, this study has several strengths, as well. Firstly, this study was conducted based on a nationwide survey, and secondly, the analyses were conducted based on gender.

## Conclusions

An interaction between SUA and TG level and BP was not observed in either men or women in our study. Moreover, high SUA level was significantly associated with SBP, while high TG level was strongly related to both DBP and SBP.

## Data Availability

Data can be accessed via https://charls.pku.edu.cn/zh-CN.

## Abbreviations

CHARLS: China health and retirement longitudinal study
CRP: C-reactive protein
FPG: Fasting plasma glucose
TG: Triglycerides
BMI: Body mass index
DBP: Diastolic blood pressure
BP: Blood pressure
SBP: Systolic blood pressure
SUA: Serum uric acid
CVDs: Cardiovascular diseases
HbA1c: Hemoglobin A1c
HUA: Hyperuricemia
eGFR: Estimated glomerular filtration rate
CDC: Centers for disease control
LDL-C: Low-density lipoprotein cholesterol
HDL-C: High-density lipoprotein cholesterol
β: Standardized coefficients
NSFC: National Natural Science Foundation of China
NIA: National institute on aging
